# Synthesis of Novel Phenyl Porous Organic Polymers and Their Excellent Visible Light Photocatalytic Performance on Antibiotics

**DOI:** 10.3390/ma12203296

**Published:** 2019-10-11

**Authors:** Xiang Gao, Jiao Liu, Zhaopeng Liu, Yuehua Deng, Wenjie Nie, Lei Zhang, Zufeng Xie, Leyuan Chen, Anning Zhou

**Affiliations:** 1 College of Geology and Environment, Xi’an University of Science and Technology, Xi’an 710054, China; LJ1379885898@126.com (J.L.); yhdeng2018@xust.edu.cn (Y.D.); nwj@xust.edu.cn (W.N.); leizh1981@xust.edu.cn (L.Z.); xzf587122@163.com (Z.X.); chenly3333@163.com (L.C.); 2 School of Chemical Engineering and Technology, China University of Mining and Technology, Xuzhou 221000, China; liuzhaopeng@cumt.edu.cn; 3 School of Chemistry and Chemical Engineering, Xi’an University of Science and Technology, Xi’an 710054, China

**Keywords:** phenyl porous organic polymers, photocatalytic, antibiotics

## Abstract

The efficient and green removal of residual antibiotics in the environment is an attractive topic. In this work, four different phenyl porous organic polymers (P-POPs) photocatalysts were successfully synthesized, and a series of techniques, such as Fourier transform infrared spectroscopy (FT-IR), scanning electron microscopy (SEM), nitrogen adsorption and desorption experimentation, and solid ultraviolet visible spectroscopy (UV-vis) were conducted to characterize the obtained P-POPs. Moreover, the photocatalytic property of P-POPs in the removal of tetracycline was studied, and the reaction conditions were optimized. Further study indicated that the P-POPs were also efficient for removing other antibiotics, such as chloramphenicol, in a high removal rate of 77%. Furthermore, the separation of the photocatalysts from the solution was easy, and the photocatalysts could be reused at least four times without a considerable loss in catalytic activity.

## 1. Introduction

Antibiotics are important drugs for the control and treatment of infectious diseases and are widely used in human medical treatment [1], livestock culture [2], aquatic culture [3] and other fields. However, after entering the environment, unmetabolized antibiotics are not only extremely difficult to degrade, but also breed other bacteria, which severely impacts the ecological balance of the area and endangers human health [4]. The removal of the residual antibiotics in the environment has become an important problem. Unfortunately, traditional antibiotic degradation technologies like the biological method [5] and adsorption [6] are often limited by their high energy consumption and high investment [7]. Therefore, it is of great significance to develop efficient and green antibiotic pollution control technology.

Photocatalytic technology can completely destroy the molecular structure of organic compounds [8] under normal temperatures and pressure, and can be used to degrade antibiotic pollution [9]. Owing to characteristics such as high treatment efficiency, mild reaction conditions, a wide application range, and rapid reaction time [10,11,12,13], photocatalytic technology has good prospects for practical application. In 1972, Fujshhima and Honda discovered that TiO_2_ could form strong radicals under light irradiation to induce photochemical reactions, and that it has a strong ability to degrade antibiotics [14]. This is the first time that humans have studied photocatalysis technology, and it is found that photocatalytic removal of antibiotic contamination is more economical, efficient, and green than other methods, but TiO_2_ as a photocatalyst can only use photons with wavelengths less than 380 nm, which is not within the visible range [15]. A number of other inorganic photocatalyst have been gradually developed for the removal of antibiotic contamination, such as CdS/ZnS [16], Bi_2_O_3_ [17], Ag/ZnO/C [18], and so on. Although much progress has been made, most of these photocatalysts need ultraviolet excitation to catalyze their reactions, which would consume much more energy and is not in agreement with green chemistry. In recent years, some inorganic-organic hybrid porous materials have been developed. For example, Sun et al. [19] synthesized g-C_3_N_4_-ZnO with excellent visible light absorption properties. Shen and coworkers [20] reported the photoactive and metal-free polyamide-based polymers for water and wastewater treatment under visible light irradiation. These photocatalysts have the advantage of having strong visible absorption and high catalytic efficiency. Therefore, the researchers turn attention to the exploration of organic, metal-free, heterogeneous photocatalysts.

As a new type of material, porous organic polymers (POPs) have the advantages of having a large specific surface area, high porosity, low skeletal density, wide synthetic route, and strong design ability [21,22]. Based on the functional and structural design ability, the introduction of an active site with catalytic function into an additional structure can form a heterogeneous catalyst with active site molecular-level dispersion [23,24,25,26]. In 2016, Li et al. [27] studied the reaction of a microporous organic polymer to catalyze the bromination of electron-rich aromatic compounds under visible light, and in 2017, Wang and coworkers [28] reported Eosin Y dye-based porous organic polymers for a highly efficient photocatalytic dehydrogenative coupling reaction. Their conclusions indicate that POPs controlled by specific functional means and morphology have an excellent ability to absorb visible light and have a high photocatalytic activity, which could pave new ways for the degradation of antibiotics.

Herein, we report the catalytic degradation of antibiotics by POPs under visible light for the first time. Firstly, four kinds of POPs with different phenyl monomers were successfully synthesized by aluminum trichloride. The resultant phenyl porous organic polymers (P-POPs) were characterized by Fourier transform infrared spectroscopy (FT-IR), scanning electron microscopy (SEM), nitrogen adsorption and desorption experimentation, and solid ultraviolet visible spectroscopy (UV-vis). Then, the photocatalytic degradation properties of P-POPs were detected, and the result show that the synthesized P-POPs are highly efficient in removing antibiotics under visible light. Moreover, the separation of the photocatalysts from the solution was easy, and the photocatalyst could be reused at least four times without a considerable loss in catalytic activity. Therefore, our study is significant from the perspective of new photocatalyst exploration, also providing some theoretical support for the catalytic removal of antibiotics by P-POPs under visible light.

## 2. Material and Methods

**Scheme 1:**
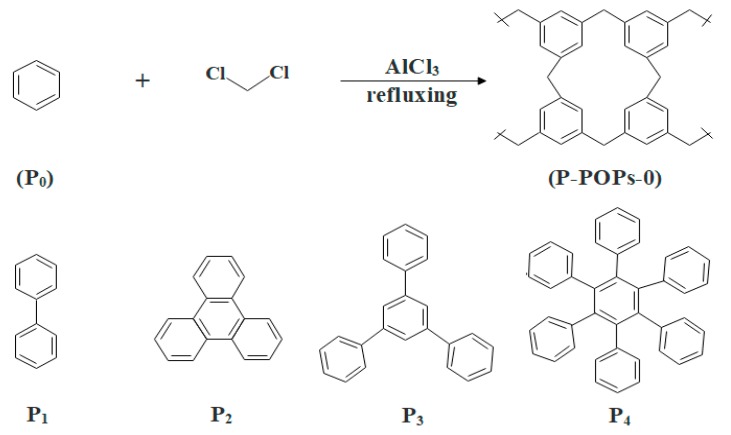
The synthetic pathway of phenyl porous organic polymers (P-POPs). P_1_, P_2_, P_3_ and P_4_ represent the structural formulae of biphenyl, triphenylene, triphenylbenzene and hexaphenylbenzene, respectively.

All reagents were of analytical grade and were not further purified. We reported the synthetic pathway of four P-POPs, as illustrated in Scheme 1 [29,30]. In one example, the preparation process of P-POPs-1 (biphenyl polymer) materials was described, and those for other materials were similar. In a typical experiment, 60 mL of dichloromethane and 1.06 g of anhydrous aluminum trichloride were sequentially added to a 100 mL double-necked round bottom flask equipped with a condenser, which was stirred at room temperature for 1 h under N_2_ protection. Then, 154.2 mg of biphenyl (1 mmol) was quickly added to the reactor and the solution was immediately discolored, then the reaction was carried out under N_2_ protection and reflux at 70 °C for 16 h. After the completion of the reaction, the resulting mixture was cooled to room temperature. The precipitate was filtered and washed twice with methanol (50 mL). Then, the precipitate was washed twice with a mixed solvent of methanol, dilute hydrochloric acid, and water (1:1:1 volume ratio) to remove unreacted aluminum trichloride. The mass of the product was washed twice with a solution of methanol and dilute ammonia (1:1 volume ratio), and finally washed with a Soxhlet wash with an aqueous solution of tetrahydrofuran and methanol (1:1:1 volume ratio) for 24 h. After the washing, the mixture was dried at 130 °C for 12 h under vacuum to obtain a biphenyl polymer material.

Four resultant P-POPs materials were observed by scanning electron microscopy (SEM, JSM-7610 F, Electronics Corporation, Tokyo, Japan). The identification of the chemical structure was conducted by Fourier transform infrared spectroscopy (FT-IR, TENSOR 27, Bruker, Germany). The nitrogen adsorption and desorption isotherms were measured at 77 K using a Micromeritics ASAP 2020M system (Micromeritics, Atlanta, GA, USA). The samples were treated at 120 °C for 24 h before the measurements. The surface areas were calculated from the adsorption data using the Langmuir and Brunauer-Emmett-Teller (BET) methods. UV-vis diffused reflectance spectra of the samples were obtained from a UV-vis spectrophotometer (UV 2600, Shmadezu, Japan).

The photocatalytic properties of the polymers were texted using the CEL-LB 70 Photochemical Reaction Chamber (Beijing Zhongjiao Jinyuan Technology Co. Ltd, China). As an example, the catalytic reaction of 20 mg/L tetracycline solution and 10 mg P-POPs-3 was described, and other reactions were similar. Next, 10 mg of P-POPs-3, 3 mL acetonitrile and 40 mL of 20 mg/L tetracycline solution were sequentially added to a 50 mL photochemical test tube. In order to uniformly distribute the catalyst in the reaction liquid and achieve the dark absorption balance, the solution was magnetically stirred for 30 min in the dark. Then, the samples were irradiated by visible light and magnetically stirred for 6 h. The photochemical reactor was irradiated with a xenon lamp (operating voltage of 50 V, current of ~6–10 A) and the ultraviolet light was filtered out. Then, the samples were irradiated by visible light and magnetically stirred. The photochemical reactor was irradiated with a xenon lamp. The sampling analysis was conducted at 1 h intervals. The absorption concentration of tetracycline was determined by a UV 2600 UV-vis spectrophotometer by recording the variations of the absorption band maximum at λ = 356 nm (TC). The removal rate (RR) was calculated by the following formula:

(1) RR=[1−(CiCo)]×100%

where *C_0_* is the initial absorbency of the antibiotic waste water solution, *C_i_* is the absorbency of the reaction solution, and RR indicates the removal rate of tetracycline in different time periods [7].

After the reaction was recovered, the photocatalyst was washed three times with an ethanol/water (1:1 volume ratio) solution. The precipitate was dried at 90 °C for 12 h under vacuum and then directly used in the next round of experiments to degrade the tetracycline wastewater. Two parallel tests were used for each cycle, and the final removal rate was averaged.

## 3. Results

### 3.1. Material Characterization

**Figure 1:**
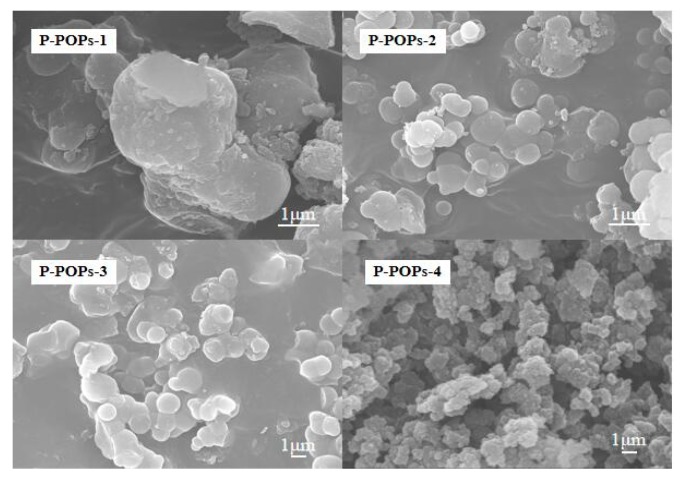
The SEM images of P-POPs-1, P-POPs-2, P-POPs-3 and P-POPs-4.

Figure 1 shows the SEM patterns of four synthesized P-POPs materials. The color of the resultant polymers varied from light yellow to brown-black depending on the different monomers and the morphology of the obtained P-POPs materials was irregular powders. As depicted in Figure 1, the average diameters of the four polymers calculated by the NanoMesurer software (version 1.2, China) package were 1.71, 0.67, 1.59, 0.08 μm, respectively, and clearly, among them, the size of P-POPs-4 was the smallest.

**Figure 2:**
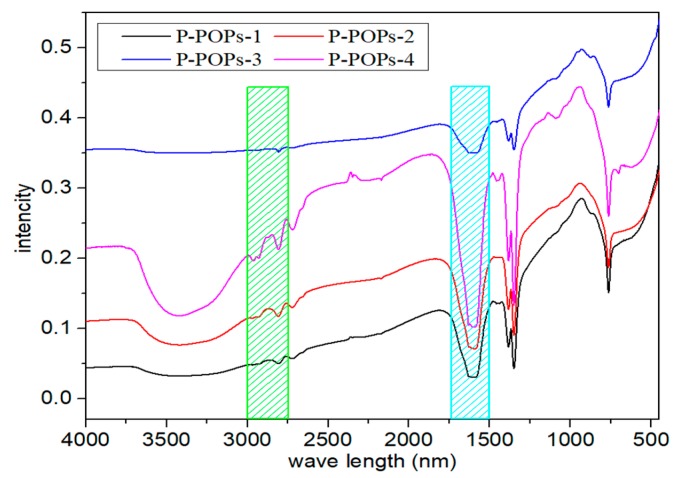
The FT-IR spectra of P-POPs-1, P-POPs-2, P-POPs-3 and P-POPs-4.

Figure 2 shows the FT-IR spectrums of four kinds of P-POPs. Clearly, there were obvious absorption peaks in the two regions of 1500–1700 nm (blue rectangle) and 2700–3000 nm (green rectangle). Therefore, based on the existence of these characteristic peaks, we could preliminarily judge that the polymers were composed of a large number of methylene groups and benzene rings as basic units. This indicted that Friedel–Crafts alkylation between the monomer and dichloromethane (as shown in Scheme 1) occurred, resulting in the presence of methylene and aliphatic carbons in the polymers.

**Figure 3:**
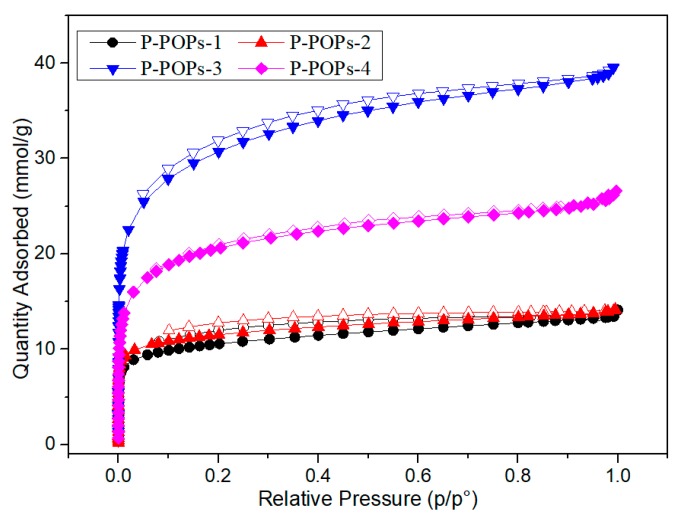
The N_2_ isotherms of P-POPs-1, P-POPs-2, P-POPs-3 and P-POPs-4 at 77K.

The porosities of the synthesized P-POPs were characterized by N_2_ sorption at 77 K. As shown in Figure 3. the four kinds of polymers materials all exhibited type I isotherms, indicating microporous characteristics. The BET specific surface areas of the P-POPs-1, P-POPs-2, P-POPs-3 and P-POPs-4 were 814, 874, 1444, and 1650 m^2^/g, respectively. The resultant polymers had large surface areas, especially P-POPs-3 and P-POPs-4, where this large surface area was possibly induced by their particular monomers owning larger molecular structures.

**Figure 4:**
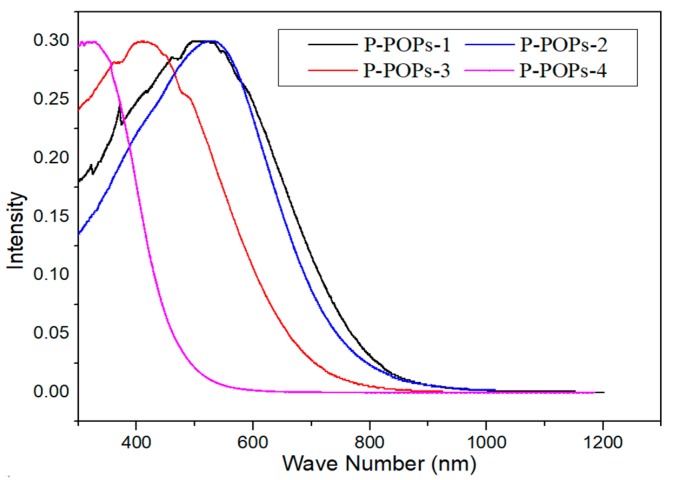
The UV-vis spectra of P-POPs-1, P-POPs-2, P-POPs-3 and P-POPs-4.

To further explore the characteristics of the resultant polymers, the UV-vis diffused reflection spectra was examined. As shown in Figure 4, from the intercept of the spectral tangent on the wavelength axis, the absorption sideband wavelengths of the P-POPs-1, P-POPs-2, P-POPs-3 and P-POPs-4 were 815 nm, 760 nm, 690 nm and 470 nm, respectively, and all were in the visible range. The band gaps of the four samples were estimated to be 1.52 eV, 1.63 eV, 1.80 eV and 2.64 eV, respectively. The P-POPs-4 sample had an obvious adsorption peak in the region of ~210–480 nm, which stated that the part of the absorption region of P-POPs-4 was in the visible light, and we also found that most of the absorption areas of the other three samples were in the visible range. Therefore, the synthesized P-POPs were all capable of photocatalysis using visible light. 

### 3.2. Activity of Catalysts

#### 3.2.1. The Effect of Different Catalysts

**Figure 5:**
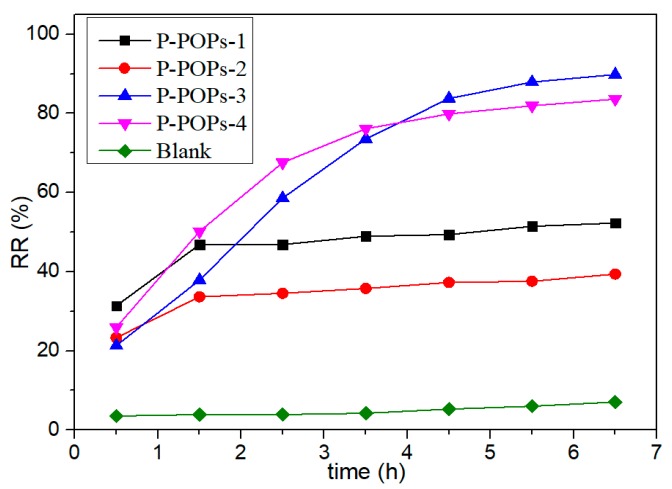
Effect of various catalysts on the removal rate of tetracycline. Reaction conditions: 20 mg/L tetracycline solution 40 mL, catalysts 10 mg, dark reaction 0.5 h, light reaction 6 h.

Since the resultant P-POPs displayed visible-light absorption, their application as photocatalytic catalysts were investigated using the degradation of tetracycline solution as a model reaction under visible light irradiation. Figure 5. shows the effects of P-POPs on the removal rates of the tetracycline solution at different times. Clearly, without the catalyst, the removal rate of tetracycline had no significant change and was only 7% after 6 h of reaction. Then, the activities of P-POPs were investigated. It was shown that these synthesized P-POPs were efficient for the degradation of tetracycline solution, meanwhile, the removal rate increased with increasing time, and a maximum was obtained at a time of 6 h. The removal rates of tetracycline by P-POPs-1, P-POPs-2, P-POPs-3, and P-POPs-4 were 53%, 39%, 90% and 84%, respectively, and among them P-POPs-3 exhibited optimal catalytic performance. This phenomenon may be due to the most suitable band gap of P-POPs-3 (the band gaps rank as follows: P-POPs-1, P-POPs-2 < P-POPs-3 < P-POPs-4), which well matched the energy of the photocatalytic oxidation of tetracycline and obtained the best removal rate. Therefore, P-POPs-3 was chosen for further investigation.

#### 3.2.2. The Effect of the Catalyst Amount

**Figure 6:**
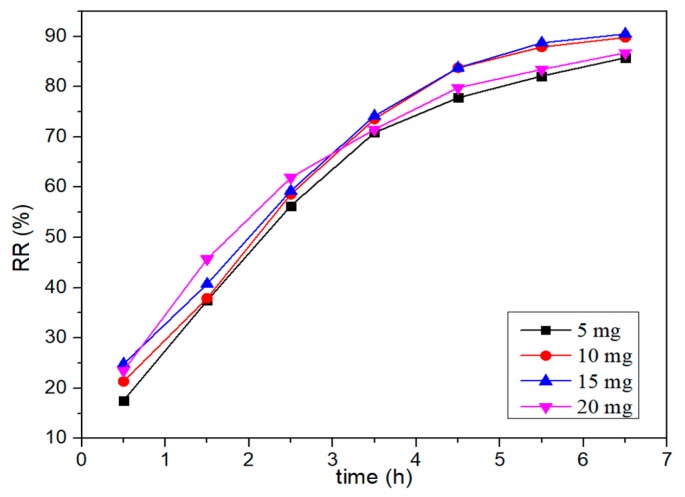
Effect of catalyst amounts on the removal rate of tetracycline. Reaction conditions: 20 mg/L tetracycline solution 40 mL, dark reaction 0.5 h, light reaction 6 h.

Figure 6 shows the influence of the amounts of catalyst on the removal rate of tetracycline under identical reaction conditions. As can be seen, the catalyst amounts had no obvious effect on the removal rate. The P-POPs-3 amounts of 5 mg and 10 mg could catalyze the reaction, giving a removal rate of 86% and 90%, respectively. This trend indicates that the catalytic efficiency increases with the amount of catalyst used. Increasing the catalyst amount to 15 mg, the removal rate achieved was 91%. In addition, we conducted the reaction with P-POPs-3 using 20 mg, and the removal rate of tetracycline unexpectedly reduced to 87%, which may be because the amount of catalyst had reached saturation. This phenomenon illustrates that continuing to increase the amount of catalyst would not increase the removal rate of tetracycline. Accordingly, the appropriate photocatalyst amount for degradation of tetracycline solution would be 10 mg.

#### 3.2.3. The Effect of Initial Tetracycline Concentration

**Figure 7:**
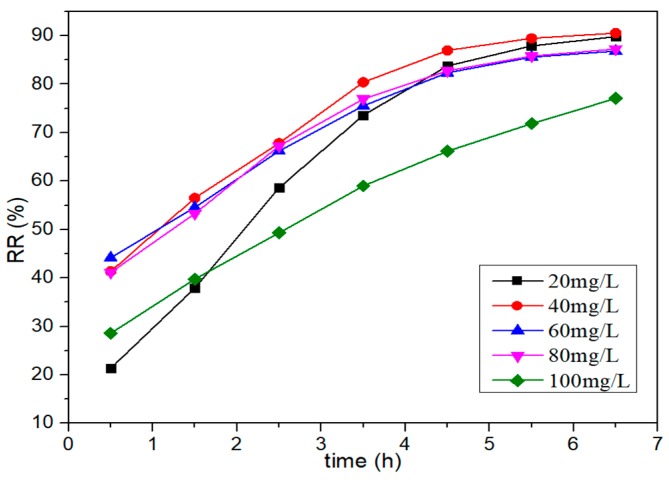
Effect of initial concentration on the removal rate of tetracycline. Reaction conditions: Tetracycline solution 40 mL, P-POPs-3 10 mg, dark reaction 0.5 h, light reaction 6 h.

In order to test the practical application of the material, the removal rate of tetracycline by the polymer photocatalyst at a different initial concentration was investigated. As shown in Figure 7. when the initial concentration of tetracycline was 20 mg/L and 40 mg/L, their removal rates were the highest, reaching 90%. When the initial concentration was increased to 80 mg/L, it still had a good removal effect of 87%. Meanwhile, a good removal rate of 77% was still obtained even when the initial concentration of tetracycline solution was increased to 100 mg/L. When the initial concentration exceeded 80 mg/L, the removal rate would decrease with increasing concentration. It was easily understandable, when the initial concentration of tetracycline was too large, the photocatalyst surface could not absorb sufficient visible light and the photocatalytic reaction rates reduced, according to the lower removal rate. The results indicated that the P-POPs-3 could be adapted to different concentrations of tetracycline even at high concentrations of 100 mg/L and exhibit good removal effect.

### 3.3. Different Substrates

**Figure 8:**
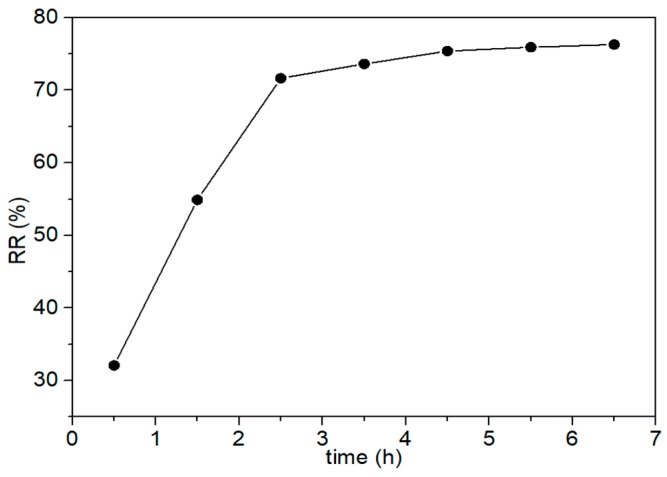
Removal of chloramphenicol. Reaction conditions: 20 mg/L chloramphenicol solution 40 mL, P-POPs-3 10 mg, dark reaction 0.5 h, light reaction 6 h.

To explore the reaction scope, the degradation of other antibiotics like chloramphenicol was examined by P-POPs-3 as the catalyst under the identical reaction, and the result was shown in Figure 8. Clearly, under visible light, the removal rate of chloramphenicol increased with increasing time under the action of P-POPs-3 photocatalyst, and a sharp increase was observed at the first 2 h, where the removal rate of chloramphenicol reached 72%. Further increasing the reaction time, the removal rate of chloramphenicol had a relatively steady increase. When the reaction time was prolonged to 6 h, a removal rate of 77% was obtained. This indicates that the photocatalytic reaction rates could increase with increasing time, however, when reaching a certain point, the growth trend remains basically unchanged. The results show that the polymer photocatalysts also had excellent removal effects on different kinds of antibiotics. 

### 3.4. Catalyst Recyclability

**Figure 9:**
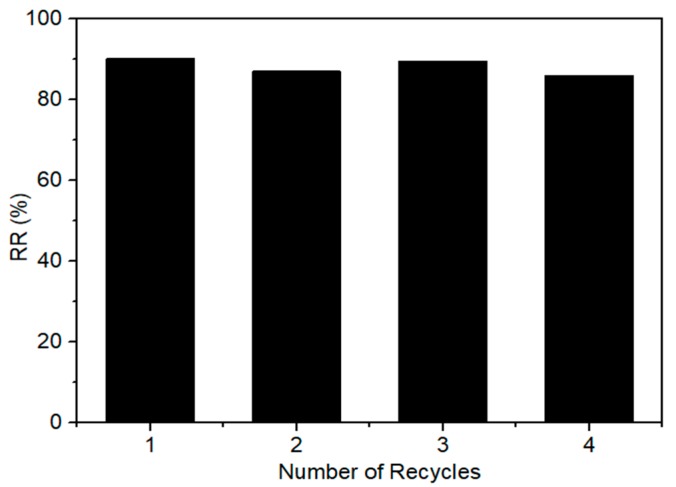
The recyclability of the P-POPs for the removal of tetracycline. Reaction conditions: 20 mg/L tetracycline solution 40 mL, P-POPs-3 10 mg, dark reaction 0.5 h, light reaction 6 h.

The reusability of P-POPs-3 was also examined for the degradation of tetracycline under the optimized reaction conditions and the results were shown in Figure 9. After the reaction, P-POPs-3 was separated from the product and washed with an aqueous ethanol solution, and then P-POPs-3 was used directly for the next run after drying. Here, the detailed procedure was the same as described in the experimental section. The removal rate of the first test was 90%, and the final removal rates of the next three cycles were not significantly reduced, and were 87%, 90% and 86%, respectively. Obviously, there was no significant change in the removal rate of tetracycline in the four cycles of photocatalyst and this phenomenon indicated that the structure and composition of the photocatalyst was not changed significantly after repeated use. The results in Figure 9 illustrate that P-POPs-3 had high stability and reusability, where it could be reused at least four times without a considerable loss of catalytic activity.

## 4. Conclusion

In this study, a method for the preparation of phenyl porous organic polymers (P-POPs) was proposed. The morphology, elemental composition, porosity, and light absorption properties of the resultant P-POPs have been studied. The results have shown that P-POPs are novel materials with good visible light absorption performance. Among them, P-POPs-3 exhibited excellent photocatalytic performance for the removal of tetracycline under visible light irradiation. When the initial concentration of tetracycline solution was increased to 100 mg/L, P-POPs-3 still had a good removal effect of 77% when only using a 10 mg amount. Moreover, P-POPs could also be applied for the removal of other antibiotics, such as in chloramphenicol wastewater, with good removal effect. In addition, the P-POPs had high stability and reusability, being reusable at least four times without a considerable loss in catalyst activity.
